# Photobleaching Imprinting Enhanced Background Rejection in Line-Scanning Temporal Focusing Microscopy

**DOI:** 10.3389/fchem.2020.618131

**Published:** 2020-12-17

**Authors:** Chaowei Zhuang, Xinyang Li, Yuanlong Zhang, Lingjie Kong, Hao Xie, Qionghai Dai

**Affiliations:** ^1^Department of Automation, Tsinghua University, Beijing, China; ^2^Department of Precision Instrument, Tsinghua University, Beijing, China; ^3^Beijing National Research Center for Information Science and Technology, Beijing, China; ^4^Institute for Brain and Cognitive Science, Tsinghua University, Beijing, China; ^5^Beijing Laboratory of Brain and Cognitive Intelligence, Beijing Municipal Education Commission, Beijing, China

**Keywords:** temporal focusing microscope, photobleaching imprinting, background rejection, biomedical imaging, two-photon effect

## Abstract

Compared with two-photon point-scanning microscopy, two-photon temporal focusing microscopy (2pTFM) provides a parallel high-speed imaging strategy with optical sectioning capability. Owing to out-of-focus fluorescence induced by scattering, 2pTFM suffers deteriorated signal-to-background ratio (SBR) for deep imaging in turbid tissue, Here, we utilized the photobleaching property of fluorophore to eliminate out-of-focus fluorescence. According to different decay rates in different focal depth, we extract the in-focus signals out of backgrounds through time-lapse images. We analyzed the theoretical foundations of photobleaching imprinting of the line-scanning temporal focusing microscopy, simulated implementation for background rejection, and demonstrated the contrast enhancement in MCF-10A human mammary epithelial cells and cleared Thy1-YFP mouse brains. More than 50% of total background light rejection was achieved, providing higher SBR images of the MCF-10A samples and mouse brains. The photobleaching imprinting method can be easily adapted to other fluorescence dyes or proteins, which may have application in studies involving relatively large and nontransparent organisms.

## Introduction

Two-photon fluorescence microscopy has become a powerful tool in biomedical deep-tissue imaging for its advantages in high spatial resolution, deep penetration, and optical sectioning capability (Denk et al., 1990; Zipfel et al., 2003; Helmchen and Denk, 2005). In biological two-photon microscopy, the fluorophore absorbs two infrared photons simultaneously to generate an emission photon in the visible spectrum. Conventional two-photon fluorescence microscopy employs the point-scanning scheme, in which all the voxels are excited sequentially and the fluorescence is detected by bulk semiconductor photodetectors. The two-photon excitation intensity is a quadratic function of the excitation radiance, so fluorescence is effectively excited only in the perifocal region (Zipfel et al., 2003). Besides, tissue scattering decrease monotonically as the wavelength decreases in 350–2,000 nm. As a result, two-photon laser scanning microscopy processes high signal-to-background ratio (SBR), which is vital in biological fluorescence imaging.

Due to the point scanning strategy, the imaging speed of the two-photon microscopy is limited by the inertia of mechanical scanners and the laser repetition rate (Kong et al., 2015). To solve this problem, temporal focusing microscopy (TFM) has reportedly achieved parallel excitation in samples. Different from spatially focusing to generate high peak intensity at the focus in the two-photon microscopy, the TFM controls the temporal profile of the pulse, whose width is minimal at the focal plane and stretches out of the focal plane. Because the excited fluorescence is inversely proportional to the pulse width in two-photon excitation process, the fluorescence intensity reaches its peak value at the focal plane (Oron et al., 2005; Durst et al., 2008; Dana and Shoham, 2011; Oron and Silberberg, 2015). Therefore, TFM achieves optical sectioning capability and, in a parallel manner, excites the full region of interest simultaneously.

According to the shape of the focus, TFM is classified into two categories: wide-field temporal focusing microscopy (WTFM) illuminates the whole 2D plane simultaneously (Papagiakoumou et al., 2010; Cheng et al., 2012; Rowlands et al., 2017); line-scanning temporal focusing microscopy (LTFM) produces a linear focus and sweeps this line in the focal plane (Tal et al., 2005; Dana et al., 2014; Lou et al., 2018). Although WTFM has a larger illuminated area, the two-photon excitation efficiency is decreased severely, according to power-law dependence on light intensity in multiphoton processes (Dana et al., 2013). Benefiting from both spatial focusing and pulse width modulation, LTFM has superior performance in imaging speed, field-of-view, depth, and axial confinement, so it has been shown to have wide applications in large-scale imaging of biological dynamics (Li et al., 2017; Park et al., 2017).

Unfortunately, as the imaging depth increases, tissue scattering distorts the excitation focus of LTFM. In turbid specimens, the power of ballistic photons *P*_*b*_ is characterized by Beer's law *P*_b_ = *P*_0_exp(−*z*/*l*_s_), where *l*_*s*_ is the effective attenuation length (EAL) and *z* is the depth (Wang et al., 2018). As the axial confinement of LTFM is corrupted in deep tissues, the out-of-focus fluorescence at different depth contributes to an intensified image background on the sCMOS detector. To solve this problem, structured illumination microscopy (SIM) enhances axial confinement and suppresses background noise by synthesizing several images illuminated by different patterns (Therrien et al., 2011; Cheng et al., 2014; Meng et al., 2017). The structured illumination pattern is only the sharpest at the focal plane, so the signal at the focal plane is extracted by reconstruction. Previous studies have reported that LTFM combined with SIM is effective to suppress the background for imaging C. elegans (Li et al., 2017). Focal modulation microscopy (FMM) can also be applied to LTFM for enhancing the SBR by subtracting an aberrated point-spread-function (PSF) image from the origin image. The background intensity is estimated from the aberrated PSF image, in which the focal intensity is decreased but out-of-focus light intensity remains. Then the background is to improve the signal to background ratio (Leray and Mertz, 2006; Zhang et al., 2018). Nevertheless, these methods need extra phase modulation devices, such as a spatial light modulator (SLM), digital micromirror device (DMD), and deformable mirror (DM), which increase the system complexity and cause laser power loss. TFM combined with sum-frequency generation also improves the axial confinement effectively but suffers from relatively low excitation power because of the partially blocking excitation beam (Durst et al., 2009).

In this paper, we proposed a novel method termed LTFM-PIM, which combines photobleaching imprinting microscopy (PIM) with LTFM to enhance axial confinement and reject the background (Li et al., 2013; Gao et al., 2014a). PIM extracts high-order fluorescence signals from photobleaching-induced fluorescence decay, and no extra device is required. We first describe the proposed method through formulations and then simulate our implementation for demonstrating background rejection improvements by LTFM-PIM. To further demonstrate the improvement of LTFM-PIM, we show imaging results of MCF-10A human mammary epithelial cells and clear Thy1-YFP mouse brains.

## Methods and Materials

**Figure 1:**
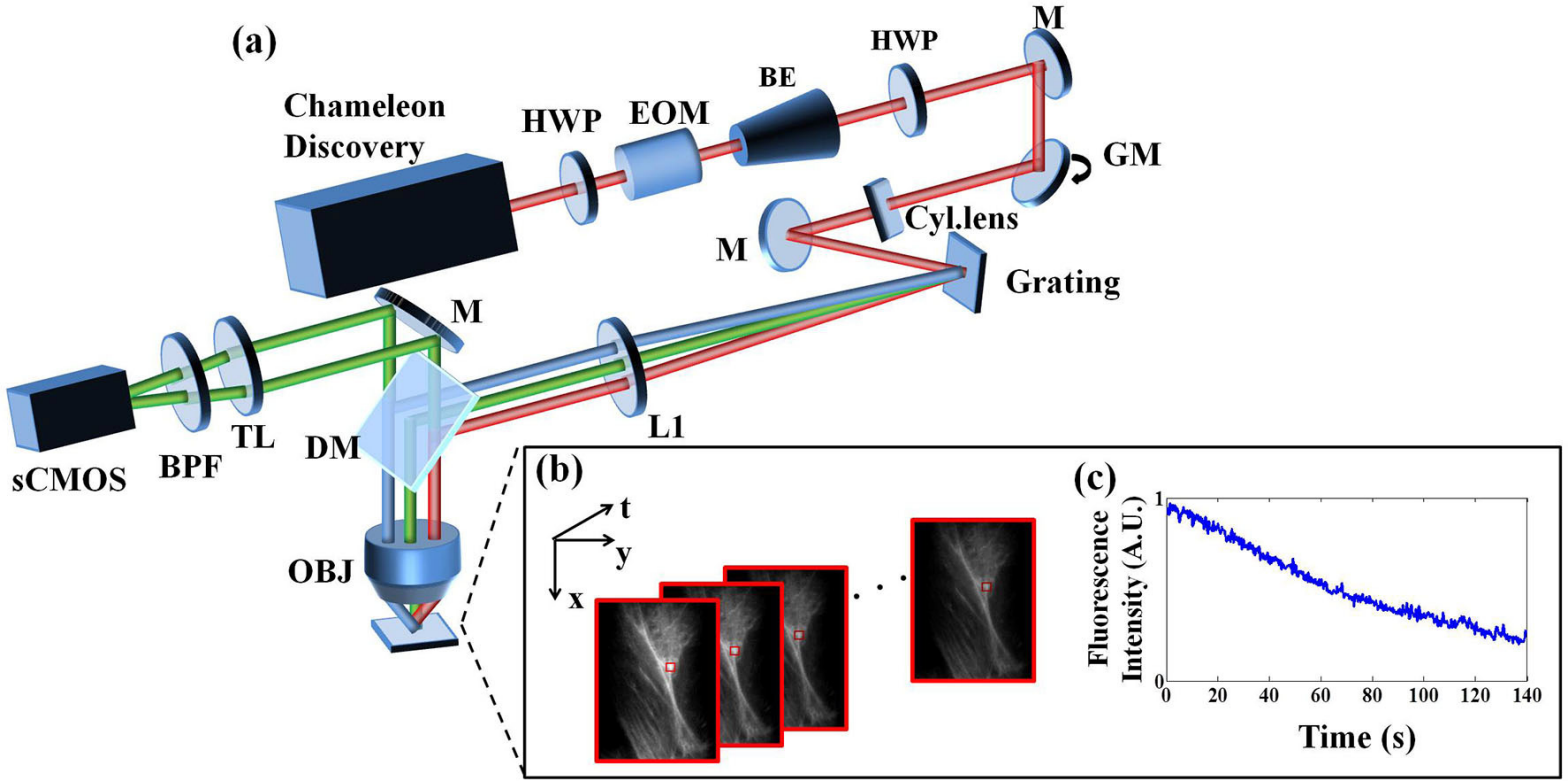
**(a)** Line-scanning temporal focusing system. The ultrashort pulsed laser was modulated in intensity by an electro-optical modulator (EOM) and formed a line-shape focus on the grating after passing through a cylinder lens (Cyl.lens). The temporal chirp induced by the grating was first broadened then compressed to the minimum at the objective focus to form axial confinement. The fluorescence image was captured by an sCMOS camera in the epidetection scheme. **(b)** A sequence of images from photobleaching imprinting microscopy (PIM). **(c)** The fluorescence polynomial decay of the subregion marked by the red box in **(b)**. Symbols: HWP, half-wave plate; BE, beam expander; M, reflective mirror; GM, galvanometer mirror; L, lens; DM, dichroic mirror; TL, tube lens; BPF, bandpass filter; OBJ, objective.

Our optical system is a standard LTFM, illustrated in Figure 1. The 80 MHz Chameleon Discovery (Coherent) is a laser source for two-photon excitation whose central wavelength is at 920 nm with pulse duration ~100 fs. The laser power was controlled by an electro-optical modulator with the polarization modulated by a half-wave plate. A beam expander (BE05-10-B, Thorlabs) was utilized to expand the laser beam to 8 mm. After this expansion, the beam was scanned by a one-dimensional galvanometer (GVS211, Thorlabs) vertically and then formed a line on the surface of the grating (830 lines/mm, Edmund Optics) after the use of a cylindrical lens (*f* = 400 mm). A half-wave plate was placed in front of the galvanometer to maximize the efficiency of grating diffraction. To ensure that the central wavelength of the 1st diffraction light is perpendicular to the grating surface, the incident beam was directed at a ~50° angle by a reflective mirror. A collimation lens (*f* = 200 mm) and an objective with NA 1.05 (XLPLN25XWMP2, Olympus), forming a 4*f* configuration, refocused the beam to a line-shape pattern on the specimen. For each scanning period, we captured a fluorescence image using an epifluorescence setup including a dichroic mirror (DMSP750B, Thorlabs), a bandpass filter (E510/80, Chroma), a 200-mm tube lens (TTL200-A, Thorlabs) and sCMOS (pixel size 6.50 μm, Andor Zyla 5.5 plus). Hardware synchronization was realized using a multi-functional DAQ (USB-6363, NI Instrument), with three voltage output channels controlling the response of the electro-optical modulator, galvanometer scanner, and camera acquisition respectively.

### Photobleaching Imprinting of LTFM

In LTFM combined with a wide-field detection strategy, the background light consists of two parts: one part is the excited fluorescence out of the focal plane (*E*_exc_), and the other part is the scattered fluorescence from the focal plane (*E*_det_). Here, we show that the PIM can suppress the out-of-focus fluorescence (*E*_exc_) and enhance the signal-to-noise ratio.

Based on LTFM, the time-averaged fluorescence photon flux <*F*>, measured by a wide-field microscope, is the integration of the fluorescence emitted from all depths (Gao et al., 2014a):

(1) $$\begin{array}{r} \langle F\left(x,y\right)\rangle =C\int \left\{\mu_{\text{a}}\left(x,y,z\right)I^{2}\left(x,y,z\right)\right\}*\text{PS}{\text{F}}_{z}\left(x,y\right)\text{d}z \end{array}$$

where *C* is a constant, μ_a_ is the two-photon absorption coefficient of the fluorophore, and PSF_z_ is the PSF of the fluorescence imaging system at depth *z*. *I* is the equivalent excitation fluence: $I\left(x,y,z\right)=\sqrt{\langle {\left[I_{\text{b}}\left(x,y,z,t\right)+I_{\text{s}}\left(x,y,z,t\right)\right]}^{2}\rangle }$, which consists of the ballistic excitation intensity *I*_b_ and scattered excitation intensity *I*_s_. The operator ^*^ represents 2D convolution and <· > represents time average.

**Figure 2:**
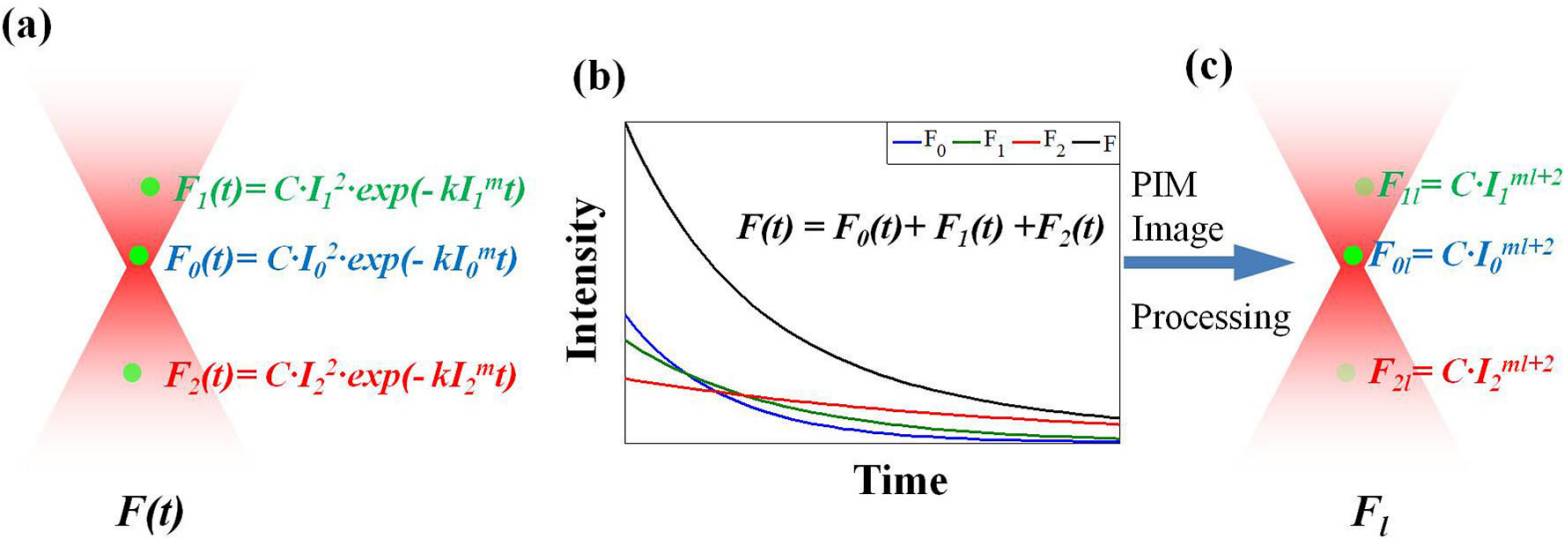
The operating principle of LTFM-PIM. **(a)** The fluorophore sample is excited by an LTFM light along the depth axis. The photobleaching rate depends on the high order of excitation intensity *I*. **(b)** In the detection process, the total fluorescence of each fluorophore is detected. **(c)** High-order PIM component *F*_*l*_, which is dependent on the *ml* + 2 order intensity distribution, is yielded by polynomial fitting of fluorescence decay.

In fluorescence microscopy, photobleaching occurs when the fluorochrome molecules are exposed to excitation light. Fluorophores irreversibly lose their ability to fluorescence due to the photon-induced chemical damage and covalent modification. The photobleaching obeys an empirical exponential temporal decay law (Gao et al., 2014b) (shown in Figure 2A) and in the *N*th frame captured at time *t*_*n*_, the absorption rate μ is also a function of *t*_*n*_, written as:

(2) $$\begin{array}{r} \mu_{\text{a}}\left(x,y,z,t_{n}\right)=\mu_{0}\left(x,y,z\right)\exp\left(-kI^{m}t_{n}\right) \end{array}$$

where μ_0_ is the initial absorption coefficient and *k* is a constant. In the case of two-photon excitation, the photobleaching rate increases rapidly with *m* ≥3 (Patterson and Piston, 2000; Gao et al., 2014a). Combining Equations (1) and (2) after Taylor expansion

(3) $$\begin{array}{r} \langle F\left(x,y,t_{n}\right)\rangle =C\overset{\infty }{\underset{l=0}{\sum }}t_{n}^{l}\frac{{\left(-\text{k}\right)}^{l}}{l!}\int_{\text{-}\infty }^{\infty }\mu_{0}\left(x,y,z\right)I^{ml+2}\left(x,y,z\right)*\\ \text{PS}{\text{F}}_{z}\left(x,y\right)\text{d}z=\overset{\infty }{\underset{l=0}{\sum }}\langle F_{l}\left(x,y\right)\rangle t_{n}^{l} \end{array}$$

where

(4) $$\begin{array}{r} \langle F_{l}\left(x,y\right)\rangle =D_{l}\int_{\text{-}\infty }^{\infty }\mu_{0}\left(x,y,z\right)I^{ml+2}\left(x,y,z\right)*\text{PS}{\text{F}}_{\text{z}}\left(x,y\right)\text{d}z\\ =D_{l}I^{ml+2}\left(x,y,z_{0}\right)\int \mu_{0}\left(x,y,z\right){\left(\frac{I\left(x,y,z\right)}{I\left(x,y,z_{0}\right)}\right)}^{ml+2}*\\ \text{PS}{\text{F}}_{z}\left(x,y\right)\text{d}z \end{array}$$

and *D*_*l*_ = *C*(*-*k)*l/l!*. In Equation (4), *F*(*t*_*n*_) is a polynomial function associated with *t*_n_ and <*F*_*l*_> is the coefficient for $t_{\text{n}}^{l}$depicted. <*F*_*l*_> can be derived from the polynomial fitting of *F*(*t*_n_). To solve <*F*_*l*_> in practice, a sequence of images is required (Figure 1B) to analyze intensity associated with time (Figure 1C).

In general, *I*(*x, y, z*_0_) is the maximum intensity of all depth in the sample, where *z*_0_ is the imaging depth. When a 3D fluorescent sample is excited by LTFM along the depth axis, the photobleached fluorophores on the focal plane decay faster than those out of focus, which are dependent on *I* (Figure 2A). The polynomial fitting of fluorescent decay yields the PIM component <*F*_*l*_ >, which contains the higher order term *I*. Therefore, when *ml* + 2 >> 1, the excited fluorescence light of the focal plane can be suppressed by the higher order PIM component (Figures 2B,C).

### Simulation of LTFM-PIM

To further demonstrate the effect of eliminating the out-of-focus fluorescence light by PIM, we assume that the spatial focus of the LTFM is same as in two-photon line-scanning microscopy, while the pulse widths were stretched by the effect of temporal focusing. Thus, we simplified the line-shaped focus model of the ballistic excitation distribution by the 1D Gaussian function shown below (Theer and Denk, 2006):

(5) $$\begin{array}{r} I_{\text{b}}\left(x,y,z,t\right)\propto \frac{2P_{\text{b}}\left(z\text{,}t\right)}{\omega_{\text{b}}\left(z_{\text{f}}\right)}\exp\left(-\frac{x^{2}}{\omega_{\text{b}}^{2}\left(z_{\text{f}}\right)}\right) \end{array}$$

(6) $$\begin{array}{r} P_{\text{b}}\left(z,\;t\right)=\sqrt{\frac{2}{\pi }}\frac{E}{\tau_{\text{b}}\left(z\right)}\exp\left(-\frac{2t^{2}}{\tau_{\text{b}}^{2}\left(z\right)}\right)e^{-z/l_{\text{s}}} \end{array}$$

where *P*_b_ is the ballistic optical power through a transverse plane, *z*_0_ is imaging depth and *z*_*f*_= *z*–*z*_0_, *l*_s_ is the effective attenuation length of the excitation light. The variable ω_b_(*z*) is the beam's half-width of the ballistic light at depth *z* and shown as follows (Theer and Denk, 2006): $\omega_{\text{b}}\left(z\right)=2\sqrt{\lambda \left(z_{\text{f}}^{2}+z_{\text{r}}^{\text{2}}\right)/\left(4\pi nz_{\text{r}}+\lambda z_{\text{f}}/l_{\text{s}}\right)}$. The Rayleigh range (*z*_*r*_) is related to the beam's far-field angular spread θ_0_: $z_{\text{r}}=\lambda /\left(n\pi \theta_{0}^{2}\right)$.

Then, the scattering excitation distribution was drawn from two-photon scanning microscopy as follows (Theer, 2004):

(7) $$\begin{array}{r} I_{\text{s}}\left(x,y,z,t\right)\propto \frac{2P_{\text{s}}\left(z,t\right)}{\omega_{\text{s}}\left(z_{\text{f}}\right)}\exp\left(-\frac{x^{2}}{\omega_{\text{s}}^{2}\left(z_{\text{f}}\right)}\right) \end{array}$$

(8) $$\begin{array}{r} P_{\text{s}}\left(z,t\right)=\sqrt{\frac{2}{\pi }}\frac{E}{\tau_{\text{s}}\left(z\right)}\exp\left(-\frac{2t^{2}}{\tau_{\text{s}}^{\text{2}}\left(z\right)}\right)\left(1-e^{-z/l_{\text{s}}}\right) \end{array}$$

where *P*_s_ is the scattering optical power through a transverse plane. The beam width and pulse width of the scattering light at a different depth are $\omega_{\text{s}}\left(z\right)=\sqrt{\omega_{\text{b}}^{2}\left(z-z_{0}\right)+4z^{3}\left(1-g\right)/\left(3l_{\text{s}}\right)}$and $\tau_{\text{s}}\left(z\right)={\sqrt{\tau_{\text{b}}^{\text{2}}\left(z\right)+8z^{3}\left(g-2\right)\left(g-1\right)/\left(9c^{2}l_{\text{s}}\right)+2z^{4}{\left(1-g\right)}^{2}/\left(3c^{2}l_{\text{s}}^{\text{2}}\right)}}_{.}$, where *g* is the anisotropy factor and *c* is the velocity of light. If we consider that the pulse width widens from temporal focusing, the Gaussian temporal profile at a different depth is estimated from wide-filed temporal focusing as $\tau_{\text{b}}\left(z\right)=\tau_{\text{0}}{\left[1+\frac{z_{\text{M}}}{z_{\text{b}}}\frac{{\left(z_{0}-z\right)}^{2}}{{\left(z_{0}-z\right)}^{2}+z_{\text{M}}z_{\text{R}}}\right]}^{\frac{1}{2}}$and$z_{\text{M}}=\frac{2f^{2}}{ks^{2}}$,$z_{\text{R}}=\frac{2f^{2}/k}{s^{2}+\alpha^{2}\Omega^{2}}$,$z_{\text{B}}=\frac{2f^{2}}{k\alpha^{2}\Omega^{2}}$ (Durst et al., 2008).

In our system, the focal length of the objective *f* is 7.2 mm, and the pulse width at focal plane τ_0_ is ~130 fs. The FWHM of each monochromatic beam $\sqrt{2\ln\;2}s$in space and the spatially-chirped beam αΩ on the back aperture of the objective are 30 μm and 3.32 mm, respectively. The effective excitation NA for spatial focusing is ~0.5, and we set the anisotropy factor *g* is 0.9. The photobleaching rate is *m* = 3, and the imaging depth is 400 μm.

**Figure 3:**
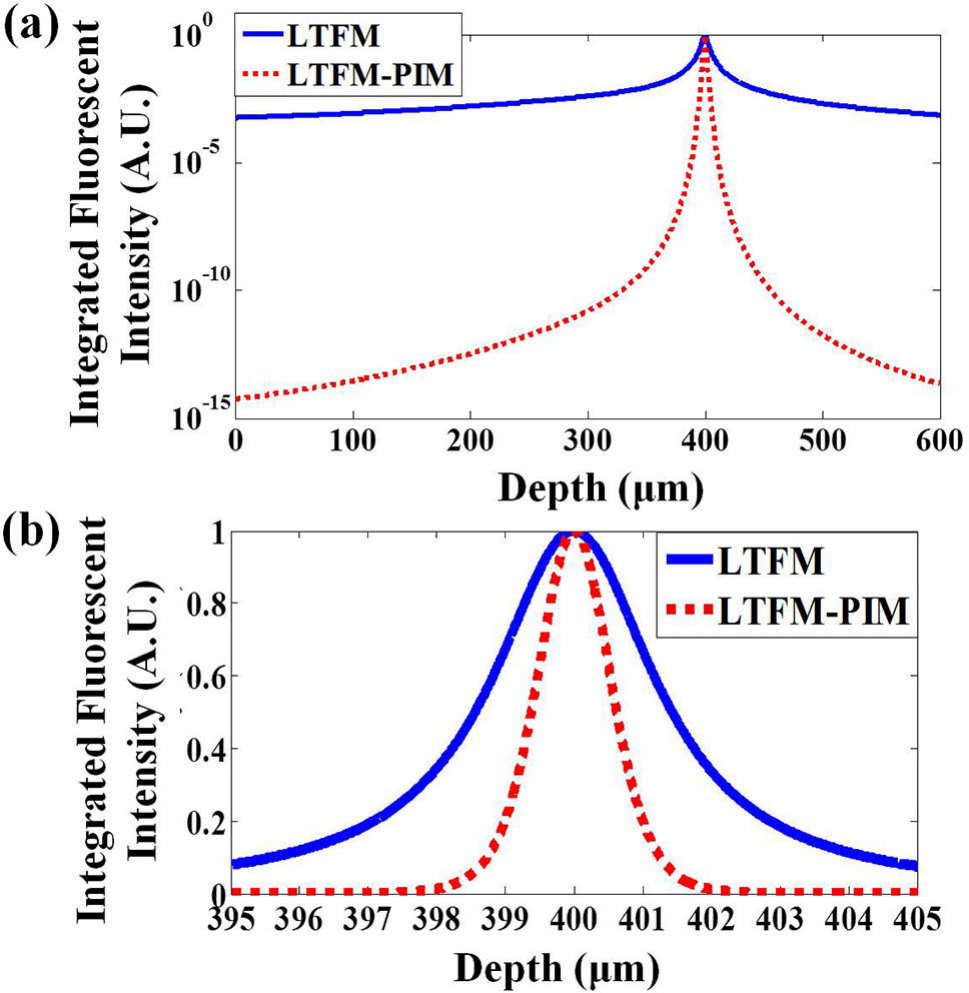
**(a)** Radially integrated fluorescence vs. depth for the LTFM and LTFM-PIM (*l* = 1). **(b)** Radially integrated fluorescence for the LTFM and LTFM-PIM (*l* = 1) near the focus. The focal depth is 400 μm, the EAL (*l*_*s*_) are 800 μm.

To evaluate the sectioning capability of the LTFM-PIM, we simulated a uniform fluorophore slice (5 nm thickness) at a different focal depth and calculated the total fluorescence of each slice *E*(*z*) by integrating the fluorescence radially. Considering that the depth of field (DOF) in our system is approximate, $\text{D}=2n\lambda_{\text{flu}}/{\text{NA}}_{\det}^{2}\approx 1.25\;\mu \text{m}$, by applying the collection numerical aperture NA_det_ = 1.05, and the wavelength of the fluorescence λ_flu_ is 520 nm, we define the energy of the signal fluorescence *S* and background light *B* as$S=\int_{z_{0}-0.5\text{D}}^{z_{0}+0.5\text{D}}E\left(z\right)\text{d}z$, $B=\int_{-\infty }^{z_{0}-0.5\text{D}}E\left(z\right)\text{d}z+\int_{z_{0}+0.5\text{D}}^{+\infty }E\left(z\right)\text{d}z$.We analyze the fluorescence in the scattering medium (*l*_s_ = 400 μm). We integrate the intensity in each fluorescent slice and the results indicate that the SBR of the LTFM and LTFM-PIM are obtained 0.4 for LTFM and 2.3 for LTFM-PIM, demonstrates that the LTFM-PIM (*l* = 1) could suppress nearly five times the out-of-focus fluorescence (Figure 3). The results demonstrate that the out-of-focus fluorescence is almost eliminated by the PIM technology, improving the SBR effectively.

### Experimental Data Processing

For practical implementation, the LTFM-PIM image was obtained by processing time-lapse images with the following steps (Xiong et al., 2020):

1. Take *M* time-lapsed LTFM images with the same exposure time: *F*_1_, *F*_2_ … *F*_*M*_;
2. For each pixel (*x, y*) in an image, we extract an intensity curve with time *F*_*N*_ (*x, y*);
3. Following Equations (1) and (4), we apply the exponential decay function to fit the intensity curve *F*_*N*_(*x, y*). To solve the intensity curve simply, we use the logarithmic operation to get: ln(*F*_*N*_(*x, y*)) = ln (*a*(*x, y*))−*b*(*x, y*)*N*. Then, we can do a linear fit on the intensity *F*_1_(*x, y*), *F*_2_(*x, y*) … *F*_M_(*x, y*) to get ln [*a*(*x, y*)] and *b*(*x, y*) of each pixel;
4. For the *l*th order PIM component, we generate the LTFM-PIM image$F_{l}\left(x,y\right)=a\left(x,y\right)\cdot b^{\text{l}}\left(x,y\;\right)$.

### Sample Preparation

#### MCF-10A Cells

MCF-10A cells (ATCC CRL-10317) were maintained and subcultured in MEBM basal medium supplemented with bullet kits (CC-3150, Lonza). 1.00 × 10^4^ cells were seeded on Φ12 mm circular coverslips (0.17 mm thickness, Fisher Brand), then fixed by 4% paraformaldehyde in the phosphate buffered saline (PBS) after overnight incubation at 37°C 5% CO_2_ incubator. Cells were permeated by 0.2% TritonX-100 for 15 min, blocked in 3% BSA in PBS for 1 h at the room temperature, and incubated with Tom20 antibody (sc11415, Santa Cruz) overnight at 4°C. The next day, cells were washed with PBS and stained with Alexa Fluor 488 labeled goat-anti-rabbit IgG (ab150077, Abcam) for 2 h at the room temperature.

#### Cleared Mouse Brain

After deeply anesthetized and perfused with phosphate buffered saline, we remove the brain from the mouse and cut it to 1 mm transverse slices after fixation. The slices are sequentially dehydrated in a series of tert-butanol (30, 50, 70, 80, 90, 96, and 100%, 2 h each step) at room temperature. The dehydrated slices are incubated in BABB-D4 (BABB: benzyl alcohol/benzyl benzonate = 1/2, BABB-D4: BABB/diphenyl ether = 4/1) for more than 1 h at room temperature until they became optically transparent. The EAL (*l*_s_) of the brainslice is approximate 800 μm.

## Results

### Imaging of the MCF-10A Sample

**Figure 4:**
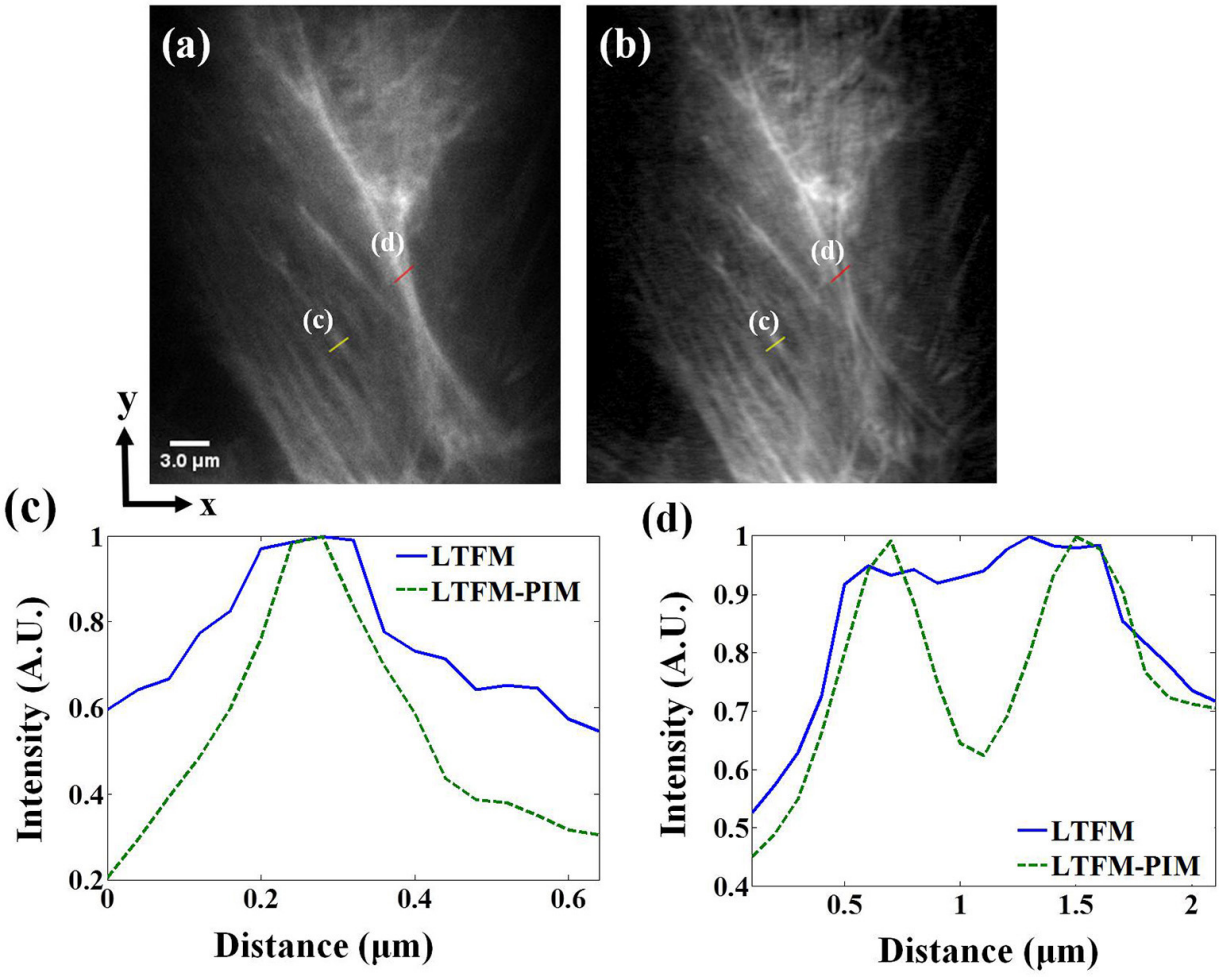
**(a,b)** LTFM and LTFM-PIM images of an MCF-10A human mammary epithelial cell, respectively. **(c,d)** Comparison of signal intensities along the yellow and red solid lines in **(a,b)**. Gamma correction 0.5 was applied to enhance the visibility of mitochondria structures.

We first validated the contrast enhancement on biological samples by imaging MCF-10A human mammary epithelial cells, whose F-actin is stained with Alexa Fluor 488. The photobleaching rate *m* of Alexa Fluor 488 in cells is 2 <*m* <4 (Chen et al., 2001). The imaging depth was ~100 μm. Fluorescence images obtained by LTFM and LTFM-PIM (*l* = 1) were presented in Figures 4a,b, respectively. The power after the objective was 200 mW. Due to the scattering light and relatively thick optical section, the in-focus structure was obscured by out-of-focus fluorescent light so that the image was blurred. By contrast, LTFM-PIM image, which was calculated by 100 time-lapse LTFM images with 0.3 s frame integration time, had a significant signal-to-background ratio (SBR) compared with the original one. We measured the intensity fluctuations alone the yellow line in Figures 4a,b, indicating that the structures were shaper in the LTFM-PIM image due to the decreased background. The SBR level of the microtubule labeled by the yellow line, labeled by (c), decreased from 0.6 to 0.2, shown in Figure 4c, owing to the elimination of the background around the microtubule. Furthermore, comparing intensity fluctuations alone the red line, labeled by (d) in each image, the signals of the microtubules were almost submerged in the background so that the closely distanced microtubules could be distinguished—see the blue solid curve in Figure 4d. After using PIM computation, we found that closely distanced microtubules were distinguished clearly with our LTFM-PIM. The background levels between the microtubules are decreased from 0.9 to 0.6 by our method—see the green dashed curve plotted in Figure 4d.

### Imaging of the Cleared Thy1-YFP Brainslice

**Figure 5:**
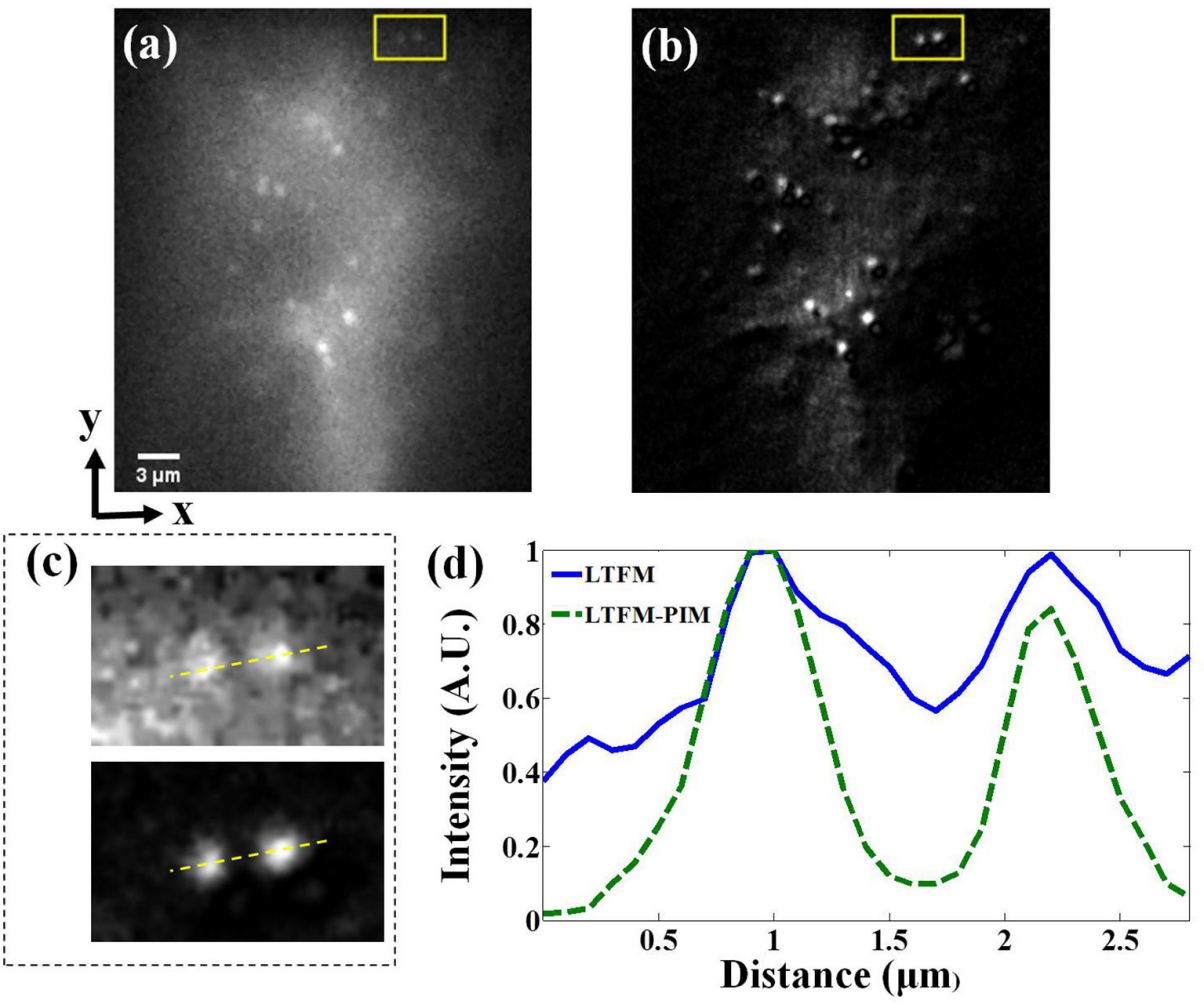
**(a,b)** LTFM and LTFM-PIM image of a cleared brainslice of the Thy1-YFP mice, respectively (about 300 μm under the surface of the slices). **(c)** Magnified view of the subregion marked by the yellow box—upper image for **(a)** and lower for **(b)**. **(d)** Signal intensity along the yellow dash lines in **(c)**.

Next, we imaged the cleared brainslice of Thy1-YFP-H mice (JAX No. 003782) to further demonstrate the contrast-enhanced performance of our technique for deep imaging. The photobleaching rate *m* of YFP in cells is >3.5 (Chen et al., 2001). The power after the objective was 250 mW. At ~300 μm under the surface of the brainslice, the fluorescence signals of the dendritic spines were almost submerged in background light because of scattering, as shown in Figure 5a. Especially in the subregion marked in the yellow box in Figure 5a, the dendritic spines were hardly distinguishable, as shown in the upper image in Figure 5c. The minimal background noise between the two spines was about 0.6—see the blue solid line in Figure 5d. By contrast, in the corresponding LTFM-PIM image (*l* = 1) which was calculated by 250 time-lapse LTFM images with 0.3 s frame integration time, the dendritic spines were visible clearly, and most out-of-focus light was suppressed. Also, compared with the same region marked by a yellow box in Figure 5b, LTFM-PIM provided a higher contrast image, allowing clear visualization of dendritic spines. The minimal background noise between the two spines was <0.2, shown by the green dashed curve in Figure 5d. The result indicated that LTFM failed to provide enough contrast, while the LTFM-PIM improved contrast approximately five times so that dendritic spines were distinguishable. Therefore, LTFM-PIM showed superior contrast-enhanced ability in the scattering medium.

## Discussion

Tissue scattering deteriorates image quality from two aspects: out-of-focus light contributes to the majority of background, while in-focus fluorescence induces image blur. We had validated the fact that the PIM could effectively eliminate fluorescence out of the focal plane (*E*_exc_), while the fluorescence crosstalk between neighboring pixels from the focal plane (*E*_det_) could be further alleviated by deconvolution algorithms (Zhang et al., 2019). In our experiment, we record photobleaching decay of the samples for tens of seconds to achieve better restriction results. To record biological dynamics such as neural activity in cells, this method could be optimized with a higher pulse energy laser and combined with novel fluorescence proteins like GFP with reversible photobleaching (Sinnecker et al., 2005; Gao et al., 2016; Niu et al., 2019).

## Conclusion

In summary, we utilized the photobleaching imprinting technique to reject background light and improved contrast by fully using the properties of line-scanning temporal focusing microscopy. With the removal of the background light, the proposed method could achieve high contrast imaging both in the transparent and scattering medium. We analyzed our method numerically by simulation and validated the performance improvement in imaging MCF-10A human mammary epithelial cells and cleared mouse brainslices.

## Data Availability Statement

The original contributions generated for the study are included in the article/supplementary materials, further inquiries can be directed to the corresponding author/s.

## Ethics Statement

The animal study was reviewed and approved by the Animal Care and Use Committees of Tsinghua University.

## Author Contributions

CZ performed the experiments, analyzed the results, made the simulations, and drafted the manuscript. XL and YZ built the imaging setup and assisted with taking the fluorescence images. LK supervised the biological experiments. HX and QD conceived the original idea and supervised the project. All authors contributed to the article and approved the submitted version.

## Conflict of Interest

The authors declare that the research was conducted in the absence of any commercial or financial relationships that could be construed as a potential conflict of interest.
